# Photostability of Contrast Agents for Photoacoustics: The Case of Gold Nanorods

**DOI:** 10.3390/nano11010116

**Published:** 2021-01-06

**Authors:** Lucia Cavigli, Boris N. Khlebtsov, Sonia Centi, Nikolai G. Khlebtsov, Roberto Pini, Fulvio Ratto

**Affiliations:** 1 Istituto di Fisica Applicata Nello Carrara, IFAC-CNR, Via Madonna del Piano 10, 50019 Sesto Fiorentino, Italy; s.centi@ifac.cnr.it (S.C.); r.pini@ifac.cnr.it (R.P.); f.ratto@ifac.cnr.it (F.R.); 2 Institute of Biochemistry and Physiology of Plants and Microorganisms, Russian Academy of Sciences, 13 Prospekt Entuziastov, 410049 Saratov, Russia; khlebtsov_b@ibppm.ru (B.N.K.); khlebtsov@ibppm.ru (N.G.K.); 3 Saratov State University, 83 Ulitsa Astrakhanskaya, 410026 Saratov, Russia

**Keywords:** gold nanorods, photoacoustic imaging, photostability, coating, miniaturizing

## Abstract

Plasmonic particles as gold nanorods have emerged as powerful contrast agents for critical applications as the photoacoustic imaging and photothermal ablation of cancer. However, their unique efficiency of photothermal conversion may turn into a practical disadvantage, and expose them to the risk of overheating and irreversible photodamage. Here, we outline the main ideas behind the technology of photoacoustic imaging and the use of relevant contrast agents, with a main focus on gold nanorods. We delve into the processes of premelting and reshaping of gold nanorods under illumination with optical pulses of a typical duration in the order of few ns, and we present different approaches to mitigate this issue. We undertake a retrospective classification of such approaches according to their underlying, often implicit, principles as: constraining the initial shape; or speeding up their thermal coupling to the environment by lowering their interfacial thermal resistance; or redistributing the input energy among more particles. We discuss advantages, disadvantages and contexts of practical interest where one solution may be more appropriate than the other.

## 1. Introduction

Plasmonic particles are emerging as a versatile solution for a broad variety of unmet needs at the crossroads of biomedical optics, sensing and imaging, owing to their unique efficiency to enhance, absorb and scatter light [1,2,3]. In particular, gold nanorods stand out as a hopeful solution combining favourable photophysical and biochemical features for critical applications as the photoacoustic imaging (PAI) and photothermal ablation of cancer [4,5], where light is respectively converted into ultrasound and heat. However, in some cases, the great efficiency of photothermal conversion of gold nanorods may turn into an issue and a practical limitation, as the input energy may trigger their overheating and reshaping, and so cause their permanent deformation, before the pathway of thermal relaxation to the environment prevails. In this mini review, we present an overview of different approaches to enhance the photostability of gold nanorods for applications in PAI, either by enhancing their thermal stability by constraining the initial shape and inhibiting the onset of pre-melting through the addition of rigid or soft shells, such as self-assembled monolayers; or by speeding up their rate of dissipative cooling towards the environment by enhancing the thermal coupling at the gold/aqueous interface through a rational design of the surface-to-volume ratio of the metal core; or also by redistributing the input energy among more particles through customization of the optical source or a subtle modulation of their plasmonic lineshapes. We discuss cases where one solution may be more pertinent than the other as well as synergistic combinations that collectively target a readier penetration of plasmonic particles into the biomedical practice [6].

## 2. Photoacoustic Imaging in a Nutshell

PAI is emerging as a powerful solution to combine the best advantages of optical contrast and acoustic detection [7]. The key idea is to shine a biological sample with short pulses from a laser or an LED to generate bursts of ultrasound, and to detect that ultrasound with a passive transducer to reconstruct an image that primarily encodes the distribution of optical absorbance. Therefore, the mechanism of contrast is optical absorbance, but the image is formed by the back-projection of an acoustic wavefront, and so does not suffer from the effect of turbidity, in the first instance. The typical duration of the optical pulses is in the ns timescale, whereas the frequency of the acoustic signal depends on the lengthscale of the target object and may span from hundreds of kHz, or less, to tens of MHz, or more. Although the penetration of light still poses some limitations with respect to ultrasound imaging at the corresponding frequency [8,9], photoacoustic tomography down to several cm is a mature achievement [10,11,12,13].

At the core of PAI is a process of photoacoustic conversion that transforms an optical pulse into an acoustic signal [14]. In turn, the process of photoacoustic conversion and subsequent propagation arises from a cascade of consecutive events that include the steps of optical absorbance, photothermal conversion and heat propagation, and thermoelastic conversion and acoustic propagation. In practice, the initial step of photothermal conversion determines a heat source [W/m${}^{3}$] that takes the simple form:

(1) $$Q_{t}=\mu_{a}\times I,$$

where $\mu_{a}$ is optical absorbance [m${}^{-1}$] and *I* is optical power density [W/m${}^{2}$].

Then, the process of photothermal conversion and heat propagation is governed by the general equation:

(2) $$\rho C_{P}\frac{\partial T}{\partial t}-\nabla \cdot \left(k\nabla T\right)=Q_{t},$$

where ρ is density [kg/m${}^{3}$], $C_{P}$ is specific heat capacity at constant pressure [J/(K·kg)], and *k* is thermal conductivity [W/(m·K))].

Instead the process of thermoelastic conversion and acoustic propagation in a liquid follows the equally general equation:

(3) $$\frac{1}{v_{s}^{2}}\frac{\partial^{2}p}{\partial t^{2}}-\nabla^{2}p=Q_{a},$$

where $v_{s}$ is speed of sound, and we have introduced $Q_{a}$ as a monopole source of acoustic pressure [Pa/m${}^{2}$]. In the particular case that the monopole source of acoustic pressure originates from a process of thermoelastic expansion in a liquid, it takes the simple form:

(4) $$Q_{a}=\frac{\alpha }{v_{s}^{2}}\frac{\partial^{2}T}{\partial t^{2}},$$

where α is a pressure expansion coefficient $\alpha =\frac{\partial P}{\partial T}{|}_{V}$.

In the standard case, where the processes of optical absorbance and thermoelastic expansion occur in the same medium, and the entire process is fast enough for thermal conductivity to be negligible, i.e., k≈0, the entire phenomenon takes the common form of a wave equation:

(5) $$\frac{\partial^{2}p}{\partial t^{2}}-v_{s}^{2}\nabla^{2}p=\frac{\alpha \mu_{a}}{\rho C_{p}}\frac{\partial I}{\partial t},$$

which may enjoy an analytical solution. For instance, in the approximation that the optical pulse is delta-like and the target object is point-like [14], the photoacoustic pressure at its surface turns out to be proportional to the product of $\mu_{a}\Gamma F$, where we have introduced Grüneisen parameter $\Gamma =\frac{\alpha }{\rho C_{p}}$, and *F* is the optical fluence [J/m${}^{2}$] of the input pulse.

However, in a more general situation, such as that of our interest, where the event of optical absorbance takes place within the gold nanorods, but the process of thermoelastic expansion develops in their aqueous medium [15,16], thermal conductivity is vital, and the intensity of the photoacoustic source actually grows with the thermal contact conductance coefficient across the metal-water interface, in particular. We will come back to this concept and its consequences in Section 4.2. For now, it suffices to remark that the photoacoustic generation from a colloidal suspension of gold nanorods is a complex process, where the steps of photothermal conversion and thermoelastic expansion are weakly coupled through the rate of dissipative cooling of the optical contrast agent. In this context, we proposed that numerical simulations by multiscale methods as the Finite Element Method [15,17] represent a convenient tool to address the dependencies of critical parameters as the evolution of temperature within the gold nanorods or the efficiency of the photoacoustic conversion.

PAI has been implemented in many labs and several platforms are available on the market for preclinical as well as translational or clinical work. In general, photoacoustic systems can be divided into two general classes [18], i.e., tomography and microscopy, depending on the combination of the setups for optical excitation and acoustic detection. In practice, the tomographic setup provides that the sample be flooded with unfocussed light of high energy per pulse and low rep rate, and the image be reconstructed through an array of transducers, whereas the microscopic counterpart that the sample be raster-scanned with focussed light of low energy per pulse and high repetition rate, and the signal be retrieved with optical or acoustic resolution. In either case, the optical fluence is of a similar order of magnitude, typically in the range of several mJ/cm${}^{2}$, in order to elicit a photoacoustic signal of sufficient Signal-to-Noise Ratio (SNR). In Table 1, we summarize the available platforms in the market and their relevant parameters, with a particular focus on the optical specifications, i.e., pulse energy, pulse duration, repetition rate and range of optical wavelengths.

## 3. Contrast Agents for Photoacoustic Imaging

As we have mentioned, the primary mechanism of contrast in PAI is optical in nature. More precisely, it is often assumed that photoacoustic images encode maps of optical absorbance. However, unfortunately, other factors concur to provide contrast, such as the heterogeneity of optical fluence and Grüneisen parameter or acoustic reflections, and their consideration is an active field of ongoing research [24,25]. Here, we stick to the elementary concept that optical absorbance is the main cause of contrast in PAI. Principal sources of endogenous contrast are biological dyes as haemoglobin and melanin. The multispectral implementation of PAI to disentangle oxy and deoxy haemoglobin and work out the partial pressure of oxygen in blood vessels with 4D resolution [26] probably remains the most successful application of PAI, and has been exploited in critical contexts as cancer [27,28,29] or neonatal brain injury [30,31], to say a few. Table 2 provides a rough overview over some sources of endogenous contrast used in PAI.

The use of PAI for the investigation of biological dyes is becoming a clinical tool of much topical interest. However, it is understood that the potential of this technology may readily extend to the broader realm of generic molecular imaging by the use of molecularly-targeted optical contrast agents. At variance with ultrasound imaging, where contrast agents need to be in the micron range of size [35] and so can hardly stain biological tissue beyond body fluids, optical contrast agents may be much smaller and thus enable more applications. A typical case is the use of optical contrast agents capable to leak out of hyperpermeable blood vessels and stain the tumor microenvironment.

**Figure 1:**
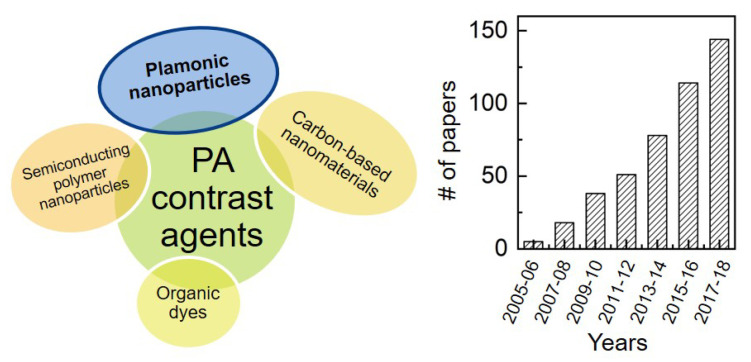
Cartoon of the various systems proposed as contrast agents in photoacoustics (on the left). On the right the trend of papers containing gold nanorod* and photoacoustic* in the topic (from ISI web of knowledge).

There exist many materials that are under investigation as exogenous contrast agents for molecular PAI, and can be divided into two general classes: inorganic materials, such as noble-metal nanoparticles and nanocarbon, and organic materials based on organic dyes, semiconducting polymer nanoparticles, etc. [36,37,38,39] (Figure 1). As a rule of thumb, organic materials often enjoy better performances in terms of biodegradability and biocompatibility. Examples include various particles incorporating organic dyes like micelles [13,40], liposomes [41] or other carriers based on proteins [42] or lipids [43], to say a few, or far red to NIR-absorbing polymers like π-conjugated systems [44], melanin [45] or polydopamine [46,47]. Conversely, inorganic systems tend to afford more ease of synthesis and manipulation, and to offer higher efficiency of photothermal conversion and higher photo- and chemical stability [39], i.e., no problems of quenching or premature leakage of dyes in biological fluids. Examples of this kind include ionic compounds like Prussian-blue nanoparticles [48,49], carbon nanotubes [50] or graphene [51], and a broad variety of plasmonic particles [52] that often consist of noble metals like gold. Among the latter class, gold nanorods are one of the most interesting and popular systems [4,53], for the reasons that will be outlined in next paragraph.

However, before we conclude this section, we briefly mention another popular direction, i.e., the use of multifunctional contrast agents for photoacoustic or multimodal image-guided interventions, such as photothermal or combinatorial therapies. Also in this case, one can find the same dichotomy between inorganic [54,55,56,57,58,59,60] and organic [61,62] nanoparticles serving as theranostic agents and often integrating multiple modules, with solutions based on gold nanorods playing a key role in the literature [63,64,65,66].

This mini review will focus on plasmonic particles, and in particular on gold nanorods. We have chosen the case of gold nanorods rather than other shapes that are being assessed as contrast agents for photoacoustic imaging, like nano-shells [67], cages [68,69] or stars [70,71], for different reasons. First, the concepts developed in this work are probably representative of a much broader class of photoresponsive materials, and so we consider the case of gold nanorods just as an illustrative example of particular prevalence in the literature. Among other models of anisotropic noble-metal particles, gold nanorods have received greatest academic and commercial interest, so much so that they are already available from major vendors like Merck Sigma-Aldrich or Creative Diagnostics. Second, gold nanorods stand out in terms of ease of synthesis and efficiency of photothermal and photoacoustic conversion, while other solutions presenting tips and dips, like nano-cages or stars, are probably best for applications based on the effect of near-field enhancement in plasmonic hot spots [72,73]. In any case, for a thorough comparison among different families of gold nanoparticles, we refer the reader to an exquisite selection of previous review [1,2,3] and research [74,75,76,77,78] papers.

### 3.1. Gold Nanorods: Many Pros and Few Critical Issues

There are many reasons why gold nanorods have become so popular as contrast agents for optical imaging, and in particular for photoacoustic applications, as confirmed by the increasing trend of number of papers over the years (see Figure 1). One of the most remarkable advantages of these particles is the possibility to precisely and easily tune their plasmonic bands across the vis-NIR spectral windows, by the simple variation of their so called aspect ratio, i.e., ratio of axial length to transversal diameter [5,79]. In simplest terms, this property can be explained by resorting to Gans theory [80], which provides for the electromagnetic solution in the case of homogeneous particles with ellipsoidal shape and complex permeability ϵ embedded in a dielectric host with real permeability $\epsilon_{m}$. In particular, the cross section for the optical extinction of randomly oriented spheroids reads Cext=4πkIm[(α1+2α2)/3], where $k=2\pi /\lambda =2\pi \sqrt{\epsilon_{m}}/\lambda_{0}$ is the wave number in the medium, and $\alpha_{1,2}$ are the electrostatic polarizabilities along and perpendicular to the symmetry axis, respectively [81]:

(6) $$\alpha_{1,2}\equiv \alpha_{\|,⊥}=\frac{V}{4\pi }\frac{\epsilon -\epsilon_{m}}{\epsilon_{m}+\left(\epsilon -\epsilon_{m}\right)L_{1,2}},$$

where *V* is particle volume and $L_{1,2}$ are the so-called geometrical depolarization factors. The localized plasmon resonance wavelength $\lambda_{LPR}$ for longitudinal excitation can be easily derived from Equation (6) and using Drude model for the dielectric function of gold [81]:

(7) $$\lambda_{LPR}=\lambda_{p}{\left[\epsilon_{ib}+\left(L_{1}^{-1}-1\right)\epsilon_{m}\right]}^{1/2},$$

where the interband contribution is $\epsilon_{ib}∼12$ and the plasma wavelength is $\lambda_{p}∼130$ nm, in the case of gold. When the geometrical depolarization factor $L_{1}$ varies from 1/3 for spheres to zero for ideal needles, Equation (7) predicts a broad variation of $\lambda_{LPR}$ from 520 nm for standard beads in water far to the infrared window.

**Figure 2:**
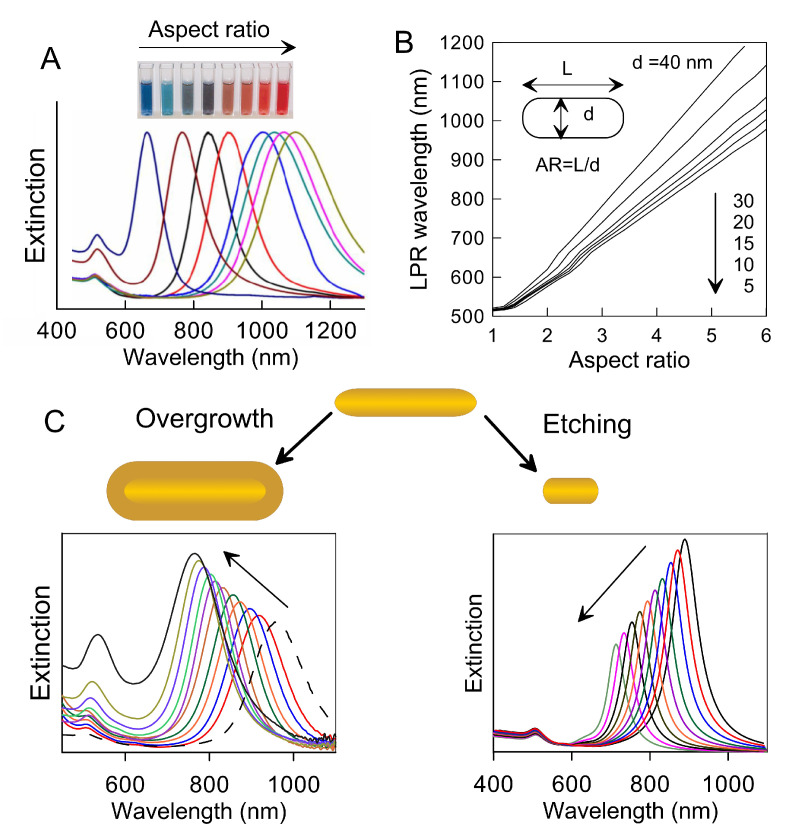
(**A**) Experimental extinction spectra of gold nanorods with aspect ratio from 2 to 7. The inset shows color images of cuvettes containing gold nanorods. Adapted by permission from Dreaden et al. [1], Copyright (2012) Royal Society of Chemistry and Wang et al. [89]. (**B**) Dependence of the longitudinal plasmon resonance wavelength on the aspect ratio of randomly oriented gold cigars of various thickness from 5 to 40 nm. Adapted by permission from Khlebtsov et al. [90], Copyright (2005) American Chemical Society (**C**) Scheme and evolution of the spectra of optical extinction during the processes of overgrowth and etching. Adapted by permission from Khlebtsov et al. [91], Copyright (2014) American Chemical Society and Khanadeev et al. [92], Copyright (2015) Springer.

Another key feature of gold nanorods is their ease of synthesis and modification. The existing protocols of electrochemical [82,83], seed-mediated [84,85,86], and templated [87,88] synthesis allow one to produce gold nanorods with length from tens to 100 nm or more and width from 5 to tens of nm. However, for the most popular seed-mediated approach and its various modifications, the possible width more typically varies within 10 to 40 nm and the aspect ratio ranges from 2 to 7. According to Equation (7), the corresponding longitudinal localized plasmon resonance wavelength of such particles ranges from about 600 nm to 1200 nm (Figure 2).

It is important to note that this range overlaps the transparency window of biological tissue of minimal absorption and scattering of light. Besides the aspect ratio, there are several other factors determining the exact position of the localized plasmon resonance wavelength. The first additional factor is shape. A prototypical rod shape is close to a cylinder with hemispherical ends (s-cylinder [90] or cigar), but other shapes such as dog-bones [93], dumbbells [94] and more complicated variants [95] are possible and recurrent as well. The shape diversity leads to a variation in localized plasmon resonance wavelength by as much as 50 nm with respect to the ideal case of cigar-like particles. The second important factor is thickness. Specifically, two sets of gold nanorods with identical aspect ratio but different thickness will display slightly different resonances, namely the localized plasmon resonance wavelength of thicker rods will be red-shifted as compared to the thinner counterpart. Figure 2B shows simulated dependencies of the localized plasmon resonance wavelength on the aspect ratio for gold nanorods with thickness varying from 5 to 40 nm. More importantly, the variation in total volume leads to significant changes in the ratio between optical absorption and scattering. For smaller particles, the optical absorption overwhelmingly prevails in the total extinction, and this is the most common case. It takes an equivolume sphere diameter as large as about 80 nm for the scattering and absorption cross sections to become equal [81], and for larger particles scattering becomes the principal pathway. Thus, tuning the aspect ratio together with the average volume allows producing effective labels for multiple applications based on diverse interactions with NIR light.

From a chemical point of view, tuning the aspect ratio and volume of gold nanorods can be achieved through the variation of the concentrations of the various reagents, and more usually the amounts of seeds and Ag ions, in a step-wise manner. However, the desired effect can be obtained by additional procedures as well. Recently, some of us suggested two useful protocols for fine tuning the localized plasmon resonance wavelength and volume of gold nanorods based on a combination of overgrowth [91] and etching [92] processes (Figure 2C). During overgrowth, the total rod volume increases and the localized plasmon resonance wavelength tends to blue-shift, together with the aspect ratio. Instead, in the etching process, the rod length undergoes a progressive decrease while the thickness remains constant. This transformation results in a progressive decrease of the localized plasmon resonance wavelength from the initial value to a final endpoint tipically around ∼600 nm. Note that etching is a fully controllable process, and the resonant wavelength can be tuned with an accuracy as good as about 1 nm. Thus, the overgrowth and etching technologies can be used for the rational design of rod geometry, and so to control the localized plasmon resonance wavelength and the ratio between optical absorption and scattering. These opportunities make gold nanorods promising candidates as labels for a wide range of applications.

In particular, the most important targets of gold nanorods are the optical hyperthermia and PAI of cancer. Since both applications derive from a process of photothermal conversion, despite fundamental differences, the rate of optical absorbance and non-radiative decay are key indicators for the performance of these particles as optical contrast agents. Various theoretical [96,97,98,99,100] and experimental [76,77,101,102] studies were carried out to quantify and compare the efficiency of photothermal conversion of different models of plasmonic particles. Electromagnetic simulations suggest that small rods enjoy the strongest absorbance in the NIR window compared to possible alternatives. On the other hand, the shape anisotropy of gold nanorods makes them very sensitive to light polarization. Experimental results of photothermal heating also demonstrate that small rods and hollow shells or cages in colloidal format yield the highest rate of photothermal conversion under NIR irradiation. It is important to remark that all mentioned experiments were carried out with low-power CW sources. For high-energy pulsed irradiation, as we shall see, the rate of heat dissipation to the environment and the phenomenon of particle photo-damage also concur in the overall process of photothermal conversion.

Another advantage of gold nanorods is their ease of modification with functional coatings. For most biomedical applications, the gold surface should be derivatized with appropriate biomolecules, such as antibodies, DNA, Raman reporters, fluorescent tags, stealth polymers, etc. [103,104]. Because of the presence of a protecting cetrimonium bromide bilayer on the surface of as-synthesized gold nanorods, their modification is often more challenging than the case of standard citrate-capped spherical beads. The use of the gold-thiol bond chemistry is a common way to functionalize gold nanorods [95,105,106], although other approaches, such as polyelectrolyte coating [107] or a pre-termination with silica [108] or polydopamine [109,110] shells are useful alternatives. In either way, such coatings play a critical role in the biological profiles of the gold nanorods, and may undertake outstanding functions, such as to enable the targeted delivery to a malignant tumor via a systemic route of administration like an intravenous injection. In this context, common strategies include the use of stealth polymers like polyethylene glycol to fool the reticuloendothelial barrier and fulfill the so-called enhanced permeability and retention effect [111,112], its association with an ingenious plethora of active solutions for host-guest recognition [113,114,115], or the integration into tumor-tropic cells serving as Trojan horses [116,117,118]. Despite remarkable progress, the targeted delivery of gold nanorods remains a lively field of ongoing research that greatly benefits from the biochemical versatility of gold.

Apart from these advantages, the work on gold nanorods also meets critical issues that still represent a major barrier against their translational penetration. In most cases, the development of contrast agents for PAI looks towards a clinical use in the human body. Without delving into the multitude of regulatory and ethical issues associated to the development of pharmaceutical compounds, we just mention that their assessment is conditional to a number of prerequisites covered under the acronym ADMET, i.e., Absorption, Distribution, Metabolism, Excretion and Toxicity. We have already anticipated that, in terms of adsorption and distribution, the scientific community has made great strides towards efficient delivery. Also the issue of toxicity has undergone extensive research with overall positive outcome [119,120]. At present, the real Achilles heel for the clinical translation of gold nanoparticles is their metabolism or excretion. In this mini review, we will overlook this key issue, while the scientific community is still summoning evidence in one direction or the other [121,122], and developing concepts for its mitigation or solution. The malicious premise is that the biochemical inertness of noble metals is an attractive feature for their stability against corrosion and nontoxicity, but may fatally work against their metabolism or excretion. We will briefly come back to this point in Section 4.2.

**Figure 3:**
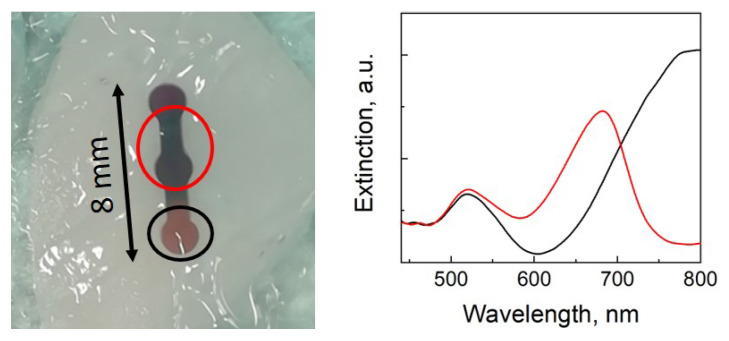
A phantom containing gold nanorods under a commercial device. On the left, a picture of the phantom after imaging under a commercial photoacoustic device for few seconds: the red oval highlights an irradiated area, while the black one shows the untreated end of the sample. On the right, the corresponding spectra of optical extinction from the selected areas are shown as lines of the same color code.

The other principal issue for the implementation of gold nanorods as optical contrast agents for PAI is their photoinstability. This problem will be the principal subject of this mini review, and, in Section 3.2, we will briefly explain why the replacement of gold nanospheres with nanorods carries along this kind of nuisance. The photoinstability of gold nanorods is not only a phenomenon of academic interest, but also a concern of very practical relevance. Indeed, gold nanorods tend to lose efficiency of photoacoustic conversion upon exposure to ns-long pulses already in a regime of optical fluence in the order of few mJ/cm${}^{2}$, more typically under 10 mJ/cm${}^{2}$, which is well below relevant permissible exposure limits at most near infrared frequencies of interest [123], and even more so with respect to the settings available in common commercial platforms for preclinical work (see Table 1), where light often shines an area in the order of some mm${}^{2}$. Therefore, the photoinsatbility of gold nanorods represents a true bottleneck in the optimization of photoacoustic applications, where the budget of optical fluence is often already quite on the edge for sufficient SNR and penetration depth. Figure 3 illustrates this problem: on the left, a phantom containing an aqueous suspension of gold nanorods displays evident signs of failure after imaging for few seconds under a commercial device optimized for preclinical work with mice (FUJIFILM VisualSonics Inc, model Vevo LAZR-X). On the right, its spectra of optical extinction reveal a substantial depletion of gold nanorods resonating at the near infrared frequencies used for imaging.

In the paragraphs that follow, we explain the origin of this phenomenon, the methods developed for a rigorous analysis of relevant thresholds, and present a roundup of possible concepts and tools proposed for its mitigation.

### 3.2. Photoinstability of Gold Nanorods: Theoretical Notions

In this section, we provide a brief overview over some of the concepts found in the literature to explain the photoinstability of gold nanorods as a process inducing their transformation into more spherical particles, and why, when and how it typically happens. Without pretence of completeness nor theoretical formalism, we provide those elementary notions that may be more useful to create the essential background and underpin the rationale behind the practical guidelines described in Section 4.

#### 3.2.1. Pre-Melting of Gold Nanorods

Even at ambient conditions [124], but more readily as temperature increases, and often even more conspicuously upon irradiation with short optical pulses, gold nanorods tend to reshape into rounder particles. The driver for this process is that the prolate shape originating from the anisotropic growth of gold nanorods is out of thermodynamic equilibrium and departs from their Wulff construction [125,126], which resembles a faceted sphere. As a rule of thumb, the larger is their aspect ratio [127] and the smaller is their size [128], the less stable is the original shape of gold nanorods. In practice, all those elements that deviate more from a stable Wulff construction, such as tips and dips, are less stable against a thermal or, of course, also a photothermal treatment.

In the case of irradiation with short optical pulses, gold nanorods tend to overheat and then to cool back down to ambient temperature through a process that entails multiple consecutive steps: the optical activation of plasmonic oscillations, Joule heating by electron-phonon coupling in a timescale below 10 ps, and then thermalization with the environment by phonon-phonon coupling in a timescale that depends on various interface features and lies in the order of hundreds of ps [127,129,130,131,132]. It is generally understood that the most of the transformation develops close to peak temperature [17], i.e., before thermalization with the environment takes over. The full transition from rods to spheres may take as short as few tens of ps [133]. These figures show well the complexity of the problem in the case of PAI. Since the most typical duration of the optical pulses implemented in all commercial platforms for PAI is in the order of few ns, the entire cascade of processes from absorption to spheroidization coexist in the time course of the optical trigger. Therefore, gold nanorods may deform and so lose efficiency of optical absorbance and photoacoustic conversion while absorbing light, i.e., well before the damaging pulse is over. Conversely, since the typical repetition rate is in the order of few Hz up to around a kHz in some cases, and thermalization with the environment takes less than about 1 ns, cumulative heating from pulse to pulse within the single particle is irrelevant. In some passages, it is also important to clarify that, with a thermal diffusivity in water in the order of 100 nm${}^{2}$/ns [134], the typical length scale involved in PAI is in the order of few tens of nm, and so thermal coupling from particle to particle is often negligible as well [135]. As a consequence, it is only the fraction of particles in resonance with the optical trigger that are at risk of reshaping [136], as will be discussed in Section 4.3. Instead, the thermal diffusivity in gold is much higher and falls in the order of 10${}^{5}$ nm${}^{2}$/ns, which is fast enough to disregard the inhomogeneity of temperature within the lengthscale of each particle [15].

The deformation of noble-metal particles does not require their temperature to reach the melting point of the respective bulk material [124,137]. Indeed, their surface-to-volume ratio is such that the dominant mechanism is pre-melting through a process of atomic surface diffusion that ends up into a collective reconfiguration by proceeding layer by layer or facet by facet without phase transition, but with thermal activation according to Arrhenius behavior [124,128,135,138]. In a recent paper [135], some of us outlined a simple model for the kinetics of the transformation of gold nanorods, where we implemented a constant value of 0.6 eV for the activation energy for atomic surface diffusion. However, an accurate description of this parameter is rather complex and subject to heterogeneity, and has been addressed in various papers. Leading principles include that its value varies with internal details, such as local curvature [128,138] or more in general coordination [139,140,141], as well as environmental features, such as stiffness [124,128]. The internal factors tend to favor the surface diffusivity of sharper tips over that of flatter plains. Instead, the environmental features modify the overall dynamics by adding viscoelastic forces that tend to constrain the initial shape. For instance, it was shown that so-called gold nanodogbones in a polyvinyl alcohol film heated to 110 ${}^{\circ }$C [125], or PEGylated gold nanorods constrained in an epoxy resin left at 120 to 220 ${}^{\circ }$C [128] failed to reach their Wulff construction, but rather converged to an intermediate shape, where the pressure exerted by the external environment compensated the effect of the prolate shape.

#### 3.2.2. Pathway to the Spheroidization of Gold Nanorods

**Figure 4:**
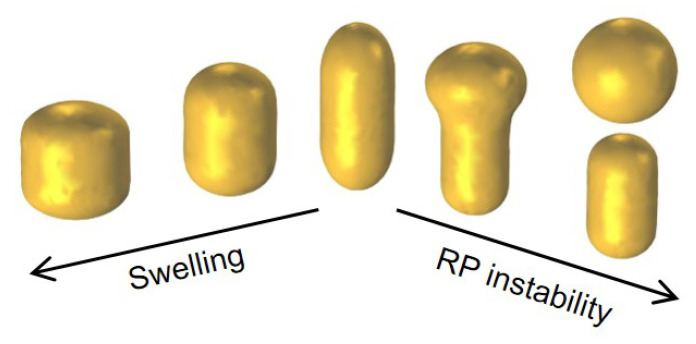
Cartoon on the principal models proposed for the spheroidization of gold nanorods based on swelling by the gradual displacement of material from the end caps to the side walls (**left** pathway) or on Rayleigh Plateau instability by the ejection of spherical fragments (**right** pathway).

From a practical point of view, the description of the exact pathway that leads to the spheroidization of gold nanorods is not as critical to the purpose of this work. However, it is interesting to note that the underlying process remains a subject of ongoing research. The fact that sharper tips are simultaneously less stable and more mobile than flatter plains has led most authors to postulate a gradual relocation of material from the end caps to the side walls of gold nanorods [128,138,142]. In the case of ns-long pulses, there may even appear a central bulge in so-called ϕ-shaped particles, as has been reported ever since earlier papers [82,143]. By causing a continuous loss of aspect ratio, this relocation would suffice to explain the common observation of a progressive blueshift of the plasmonic band of longitudinal oscillations. However, in the case of ultrasmall particles, other authors have evoked more complex phenomena, such as the ejection of spherical fragments according to Rayleigh-Plateau instability (see Figure 4) [144]. The physical principle underlying this phenomenon is that the emergence of ripples of appropriate period along the side walls of gold nanorods may dictate a gradient of surface tension capable to feed a snowball effect of continuous amplification until rupture into droplets, as occurs in metal wires [145] and, perhaps more iconically, in hydrodynamic systems as liquid columns. The consequence in this case would be a blueshift of the plasmonic band of longitudinal oscillations accompanied by a decrease of the ratio between their longitudinal and transversal components.

Finally, we mention that other processes that have been described in the literature include explosive fragmentation [129,146], sublimation [147], as well as collective pathways towards a decrease of the specific surface area of the particulate as a whole, such as coalescence and Ostwald ripening [142,144], i.e., an exchange of material from smaller or less stable to larger or more stable particles. Explosive fragmentation and thermionic emission have been conjectured to explain a certain loss of size of gold nanorods upon irradiation in a fs regime of optical pulses. Conversely, the possibility of coalescence and Ostwald ripening has been invoked in a continuous regime of thermal annealing in the presence of a large excess of surfactant [142], or in the case of ultrasmall particles [144]. As far as PAI is concerned, these extra processes probably play a negligible role, and a progressive loss of aspect ratio remains the mechanism of most importance.

### 3.3. Methods to Characterize the Instability

In this section, we present a brief overview over some of the experimental protocols reported in the literature to define and quantify the threshold fluences F${}_{\mathrm{th}}$ before gold nanorods reshape and lose efficiency of optical absorbance at the interrogation wavelength.

As already mentioned in the previous section (see Figure 3), commercial platforms for PAI unfortunately offer little control over key parameters as optical fluence. For this reason, they are not always the best tool for the optimization and performance assessment of new contrast agents, in particular with respect to photostability, where fine control over the impinging fluence is necessary to identify the damage thresholds. In order to overcome these limitations, dedicated experimental set-ups and specific protocols have been designed over the past years. However, a standardized set of guidelines and criteria is still not available. As a consequence, several values for the threshold fluence F${}_{\mathrm{th}}$ can be found in the literature, ranging from a few up to hundreds of mJ/cm${}^{2}$, with this large variability not only depending on the different properties of the plasmonic particles under investigation, but often also more substantially on the different measurement criteria as well as the phantoms used for their implementation.

One of the most popular and straightforward protocols is based on the comparison of the spectra of optical extinction of the gold nanorods before and after exposure to the laser beam for a given number of pulses at a given average fluence [63,123,148,149,150]. The onset of a depletion of optical extinction at the longitudinal plasmonic peak position, similar to those reported in Figure 3, indicates the occurrence of partial reshaping in the particle ensemble. The analysis of such losses allows for a quantitative definition of a damage threshold [123,149], but relevant values are hardly comparable from experiment to experiment. For instance, the onset of damage may depend on whether the same sample is reused for exposure at different conditions [151], or instead refreshed after each train of pulses at a given fluence [123]. This protocol has been mainly applied with liquid samples, and its implementation requires laser sources of high energy, in order to homogeneously shine large areas of cuvettes or multiwell plates containing the dispersion of plasmonic particles for investigation with standard spectrophotometers.

**Figure 5:**
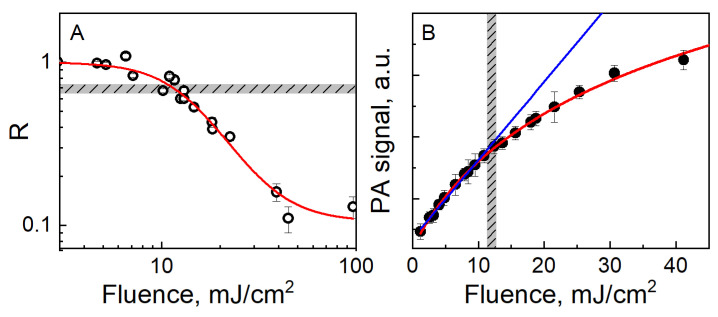
Methods to analyze the photoinstability of gold nanorods. (**A**): Typical sigmoid trend of the parameter labelled as R (see the main text for a definition) vs. excitation fluence. (**B**): Representative example for the occurrence of a sublinear trend of photoacoustic (PA) signal vs. excitation fluence, denoting the onset of reshaping.

An alternative approach exploits a photoacustic probe in the place of the spectrophotometric analysis to detect the transformation of the contrast agent after an optical excitation [17,152]. Through a photoacoustic microscope, the particle transformation is monitored by taking the ratio R of the efficiency of photoacoustic conversion from a certain spot in a phantom after/before irradiation with a train of pulses from a focused source at a given fluence. The photoacoustic signals used as reference to probe the efficiency of photoacoustic conversion are taken at a fluence significantly lower than the damage threshold. As in the case of the loss of the longitudinal plasmonic peak intensity (see Figure 5A), a decay of R from unity denotes a photoinstability. In general, the trend of R as a function of the average fluence of optical excitation starts from unity, and then exhibits a decreasing sigmoid trend, as it is typical for any population with a negative growth rate [17,123], which reflects a depletion of available particles in resonance with the irradiation. Usually, the photostability threshold is set in correspondence to a value of R that is close enough to unity to capture the very onset of photodamage, but also far enough to ensure statistical significance. A value of $\frac{3}{4}$ is often a decent choice for general purposes. This protocol is particularly suitable for semi-solid samples [17,152].

Another method that exploits the dependence of the photoacoustic response on the optical absorbance of the gold nanorods [14] is based on the dynamic analysis of the trend of the signal amplitude with the extant fluence of the optical excitation [151,153,154,155]. For each condition, the signal amplitude is averaged over a given time or number of pulses, and then added to a scatter plot as a function of optical fluence. According to the basic principles outlined in Section 2, in the absence of photodamage, the trend should keep linear with slope proportional to $\mu_{a}\Gamma$. Instead, in the presence of a photoinstability, the relevant threshold manifests as the onset of a sublinearity (see Figure 5B). When the sample is not refreshed from condition to condition, this method compares to the first case described in this paragraph, because it does not decouple from cumulative effects that may add up from pulse to pulse and depend on all details of the particular sequence and protocol in use. In order to avoid such effects, it is possible to implement a microfluidic set-up to continuously keep the sample in flow [153,154,155]. In this case, the trend captures the onset of particle reshaping within the duration of a single pulse, and the relevant threshold may differ from the previous methods. In a recent work [154,155], some of us implemented this measurement in an all-optical device suitable for the confinement of ultra-small fluid volumes, where miniaturization mitigates the requirements on the output energy delivered from the optical source for a thorough characterization of the system.

Finally, we mention the possibility for a more qualitative and expedite approach based on the continuous monitoring of the photoacoustic signal over time or number of pulses at a certain fluence of interest [60,156]. In this case, it is typically decided whether a sample is photostable or not under a particular setup, without the interest in quantifying a threshold fluence. While this approach falls a little outside of the scope of this mini review, because it is not a first choice for a rigorous analysis of the photostability of gold nanorods, it may be the only option available when using a commercial platform for preclinical or translational work, and, for instance, assessing the possibility to use an optical contrast agent in a certain experiment.

To conclude this section with a note of caution, we remark that the translational relevance of all these protocols and designs for phantoms may arouse controversy, because particles intended for use in vivo are likely to lose their colloidal stability at some point after systemic injection, for instance upon clusterization within endosomal vesicles [119,157], which may modify their overall photothermal behavior [158]. However, this doubt was addressed in a recent publication [135], which revealed the absence of substantial effects on the photostability of gold nanorods over a broad range of regimes of optical irradiation even after substantial interaction with their biological target. Therefore, the fluence thresholds measured with the methods overviewed in this paragraph are suitable to assess the photostability of gold nanorods for use as optical contrast agents in PAI. In next section, we describe some of the various strategies implemented to enhance this parameter.

## 4. Strategies to Improve the Photostability of Gold Nanorods

In view of its importance in typical application scenarios, different authors have undertaken the development of solutions to mitigate the issue of the photoinstability of gold nanorods. Here, we propose a broad classification of some of these methods chosen for their relevance and sorted according to general concepts based on our understanding of the underlying phenomena. While we are aware of the pitfalls hidden in a strict compartmentalization, our work intends to bring some order in a literature that is recent but already fragmentary, and to orient the experimentalist who wishes to consider the use of gold nanorods in PAI. In this sense, we associate each method with due references and an overview over relevant protocols for their practical implementation, and with a brief but multidimensional discussion of their pros and cons for biomedical applications.

### 4.1. Improving the Thermal Stability of Gold Nanorods: The Effect of Coating

This strategy intends to increase the thermal stability and to slow down the course of reshaping of gold nanorods by inhibiting their process of pre-melting, whether they be exposed to an optical excitation or left on a hot plate, in an oven, etc. The most common approach to this effect consists in constraining the initial shape and frustrating the process of spheroidization by the addition of rigid or soft shells. Kennedy et al. [128] have described the effect of a stiff environment, such as an epoxy resin, hosting gold nanorods as a viscoelastic medium imposing an external pressure that tends to compensate the gain in Gibbs free energy brought by spheroidization. In practice, the stiffer is the dispersion medium, the closer becomes the equilibrium configuration of the particulate system to the as-synthesized rods, rather than their Wulff construction. Of course, the dispersion of an optical contrast agent in a stiff medium is a scenario of little interest in the context of PAI. However, this notion may be implemented at a much more local scale about each particle, as the addition of rigid or even soft shells. It turns out that coating of gold nanorods may affect their thermal stability through multiple and synergistic mechanisms, as outlined hereunder.

The use of rigid shells is arguably the most popular and earliest solution implemented for the stabilization of the photoacoustic response from gold nanorods. In particular, the most common shell material is silica or organosilica, which enables fine control over parameters as thickness or the level of porosity [159]. An enhancement of the photostability and thermal stability of gold nanorods after silanization has been observed by different authors, both in a continuous regime of thermal treatment [160,161,162] and upon optical excitation with ns- [63,82,156,163,164] as well as fs- [165] long pulses. The use of silanized gold nanorods for applications in PAI has been a subject of extensive research. In addition to its effect on stability, Chen et al. [163] demonstrated that an optimal shell of a thickness around 20 nm is able to amplify the photoacoustic signal from gold nanorods embedded in a gelatin phantom by as much as a factor around 4, probably due to the enhancement of heat transfer through the interfaces between gold, mesoporous silica and water. In a more recent work [108], some of us provided a quantitative definition of the threshold fluence for the onset of photoinstability in gold nanorods modified with organosilica, which improves by something in the order of 10 % on average per nm of shell thickness, in the case of a mercaptosilane precursor. Above this threshold, failure of the shell originates from its plastic deformation and eventual rupture due to elastic stress [161,165]. In the case of mesoporous silica, some authors have even reported that the eventual breakdown of the plasmonic system may depend on a leakage of fluid gold through interconnected pores or small breakpoints [165].

In this context, the use of soft shells may be a beneficial alternative to brittle silica as well. Several authors have predicted [140] and observed [142,144,166] that a weak adsorbate may enhance the thermal stability of ultrathin gold nanowires and rods by retarding their surface atom diffusivity, and that a stronger interaction may enhance this effect. For instance, we mention the concept of polyelectrolyte coating [167,168] as a versatile approach to obtain soft shells and modulate relevant parameters as shell thickness by the adsorption of consecutive layers of oppositely charged polymers. More recently, we found that a shell of aromatic thiols as thin as about a molecular monolayer is sufficient to redouble the threshold fluence for the deformation of PEGylated gold nanorods [169]. We have associated such a remarkable effect to the combination of different factors, including the tight anchoring of the adsorbate thiols to the gold surface, their strong mutual bonding through inter-molecular hydrophobic interactions like pi stacking, and their self-healing capacity supporting continuous dis- and re-aggregation and providing resilience against deformation of the soft network. In practice, the picture would be that of a high density of thiol-pinned gold adatoms jammed in a crowded jungle of covalent and non-covalent bonds.

Finally, we mention that a possible trade-off between the extremes of hard and soft shells may be metal shells, which hold the potential for a functional combination of stiffness and plasticity. In a recent work [170], we have reported the empirical observation that gold-core/silver-shell nanorods apparently exhibit extreme thermal stability for photothermal applications, and we believe that this direction deserves additional investigation.

#### 4.1.1. Coating of Gold Nanorods with Silica and Organic Polymers: Useful Protocols

**Figure 6:**
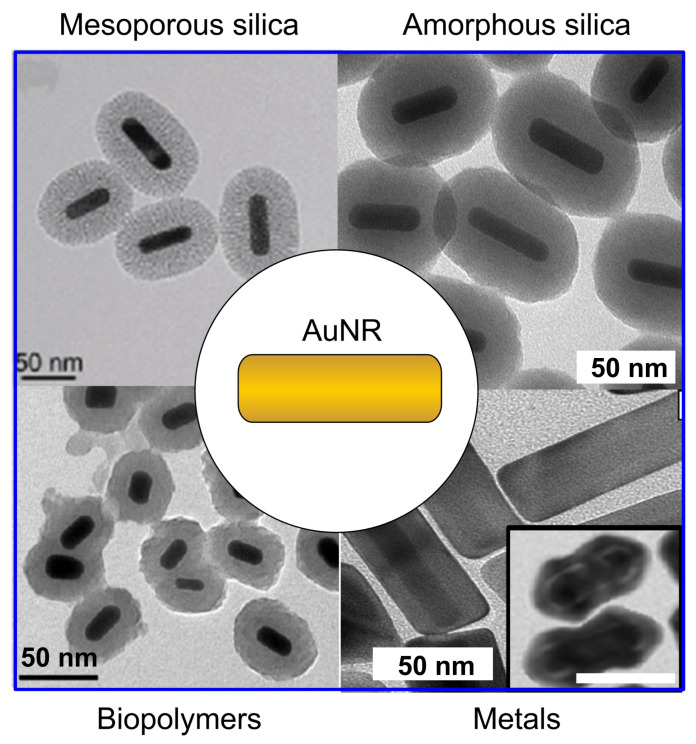
General types of gold nanorod-based core/shell particles. Adapted by permission from Wang et al. [174], Copyright (2016) American Chemical Society, Khlebtsov et al. [175], Copyright (2016) Springer, Tsai et al. [176], Copyright (2013) American Chemical Society.

A common approach to enhance the functionality of gold nanorods is the formation of inorganic or polymeric shells. A first choice in this context is the addition of silica (general types of gold nanorod-based core/shell particles are shown in Figure 6. Gorelikov and Matsuura suggested a robust and simple method to synthesize a shell of mesoporous silica with controllable thickness [171]. This method consists in the hydrolysis and condensation of tetraethyl orthosilicate in a solution containing cetrimonium bromide at pH around 10 to 11. As a result, gold nanorods are coated with a rigid shell of thickness between 5 to 30 nm and with pore size between 2 to 4 nm. Several modifications of this approach were further suggested to improve the quality of coating. In particular, the modified protocols include the replacement of the aqueous buffer with a water/ethanol mixture [172] and the formation of pores with controllable size, morphology as well as orientation [65,173]. The porous structure of silica does not only stabilize the suspension of gold nanorods, but also serves as an effective platform to add a broad variety of functional ingredients.

The protocols for the deposition of mesoporous silica often suffer from poor control over thickness. Other popular protocols to coat gold nanorods with silica derive from Stöber process in the presence of templating particles [177]. Stöber synthesis consists in the hydrolysis and condensation of tetraethyl orthosilicate in alcoholic medium. Note that as-prepared cetrimonium-coated gold nanorods cannot be suspended in ethanol or other alcohols. In order to fix this problem, Liz-Marzan and coworkers suggested a universal approach based on the preliminary PEGylation of the particles followed by the deposition of silica via Stöber process [178]. This approach is suitable for a broad variety of particles as well as a wide range of thickness of the resulting shell. Beside the preliminary step of PEGylation, other pre-coating strategies were reported by using polyvinylpyrrolidone [179] or a thin layer of silica [175,180] before suspension in alcoholic medium. In situ coating of gold nanorods with amines or thiols is also possible by using aminosilanes [181] or mercaptosilanes [108,182] alone or in combination with tetraethyl orthosilicate. From the optical point of view, coating with silica leads to a red shift of the plasmonic bands and increases the extinction and scattering cross-sections especially in a shorter wavelength region [72,183,184].

The use of organic polymers is another popular option to coat gold nanorods. Usually, the polymerization of monomers on the particle surface is a substantial challenge that conflicts with the colloidal stability of the suspension. However, polydopamine has recently been suggested as a robust and convenient coating for various kinds of particles [185,186]. Polydopamine is a black biopolymer with chemical structure and optical properties resembling those of melanin [187]. The protocols are very simple as dopamine directly polymerizes on the surface of gold nanorods under pH around 8.5 [185]. A simple variation of the ratio between monomers and gold nanorods provides exquisite control over shell thickness from 2 to 50 nm. In addition, polydopamine exhibits a convenient variety of functional groups for further conjugation [188]. As polydopamine absorbs light over a broad window, its effect on the optical extinction of gold nanorods differs from that of silica. For example, some of us recently reported an unusual decrease in the longitudinal band pf plasmonic oscillations of polydopamine-coated gold nanorods [189].

**Figure 7:**
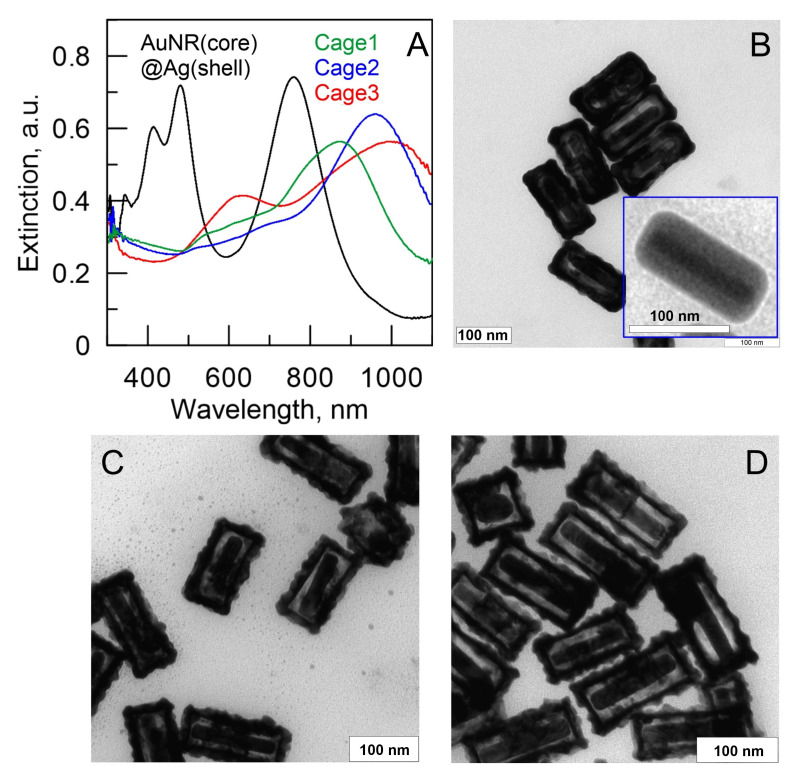
Spectra of optical extinction (**A**) and TEM images (**B**–**D**) of gold nanorod-in-shell particles, also sometimes called anisotropic nanocages. The inset in the panel B shows the initial Au@Ag nanocuboids that display multimodal extinction below 500 nm.

Finally, we also mention that gold nanorods can serve as a template for the synthesis of all-metal core/shell nanorods. The formation of uniform shells of silver on gold nanorods is a useful method to fabricate systems with new optical properties. One of the first reports on silver-coated gold nanorods with controllable shell thickness was published by Ah et al in 2011 [190]. A modified protocol was further reported by Okuno et al. [191], who improved the control over shell thickness and shape. gold-core/silver-shell nanorods exhibit much sharper, stronger, and blue-shifted plasmonic resonances with respect to their gold cores alone. Even a shell of silver as thin as 2 nm suffices to blue-shift the longitudinal band of plasmonic oscillations of gold nanorods by tens of nm [192]. In addition, rod-in-shell structures with tunable optical properties can be obtained through a galvanic replacement reaction, where the gold-core/silver-shell nanorods serve as template and a solution of chloroauric acid works as oxidant [176,193]. Figure 7 shows TEM images and spectra of optical extinction of these so-called anisotropic nanocages. By rational design of such interesting structures, it is possible to tune their optical behavior as well as additional parameters as roughness [15] or specific surface area.

In conclusion, the scientific literature provides multiple examples of protocols to coat gold nanorods with hard, soft or other shells, such as silica, organic polymers as well as newer solutions as metals, which hold the potential to enhance their photostability. The use of silica or organosilica is the safest bet for its recurrence in the literature. But the other options may offer advantages in terms of ease of synthesis and purification.

#### 4.1.2. Coating of Gold Nanorods with Silica and Organic Polymers: Pros and Cons

Coating with rigid or soft shells obviously implies a certain level of additional complexity that alters multiple features of the initial particles, including their hydrodynamic and electrokinetic profiles. Such modification may come with advantages and disadvantages but, in any case, needs understanding within an overall design of the system. Practical disadvantages include a probable complication of the procedures for purification, due to the multiplication of the steps of synthesis and a possible increase in the specific surface area of the particulate. For instance, gold nanorods modified with organosilica tend to retain a higher fraction of cytotoxic impurities used in their synthetic protocols [108], such as cetrimonium bromide, and so to exhibit more cytotoxicity. With respect to silica, coating with biopolymers as polydopamine is an excellent strategy to gain atoxicity [194,195]. Another obvious difference relates to all those applications that require direct access to the noble-metal surface, such as various methods for optical sensing based on Surface-Enhanced Raman Scattering [196,197] or localized Surface Plasmon Resonance [198] shifts that may complement the photoacoustic functions. Not that these methods become impossible [199,200,201], but their use needs thorough rethinking based on the presence of a spacer, holder or a porous filter probably extending beyond the typical reach of near-field components.

The most obvious advantage of a strategy based on the enhancement of the thermal stability of gold nanorods is a simultaneous protection of both channels of photoacoustic as well as photothermal conversion [169], at variance with the approach discussed in next section. In the case of porous shells, another attractive feature is their ease to host additional ingredients, such as drugs, photosensitizers and reporters. Both silanized [65,202,203,204,205,206] and polyelectrolyte-coated [207,208] gold nanorods have become a popular choice to deliver and controllably release drugs and genes upon photothermal activation with exquisite control. The recent literature contains a plethora of examples of systems for multimodal therapy of cancer integrating for instance photothermal ablation and chemotherapy within the scope of the same particles [209]. Another popular idea is to enrich silanized and polyelectrolyte-coated gold nanorods with dyes for use in multimodal imaging, such as photoacoustic and fluorescent [210], photoacoustic and Raman [211], photoacoustic and non-optical methods as MRI [212,213], or other applications that combine well with a hyperthermia treatment, such as local thermometry [203]. In some cases, organosilica [108] or polydopamine [110] may just represent a convenient cross-linker for particle construction. Another advantage of rigid shells as silica is that their steric hindrance provides a reliable solution against plasmonic coupling in the case of particle aggregation in a biological environment [108,158], for instance after endosomal uptake. Plasmonic coupling stands alongside the issue of photoinstability as another major threat to the optical stability of gold nanorods [158].

Overall, the addition of rigid of soft shells to mitigate the photoinstability of gold nanorods used as optical contrast agents for PAI is an effective approach that makes particular sense in a context where the the implementation of this solution belongs to a multidimensional rethink of the particles and their functions.

### 4.2. Lowering the Thermal Resistance at the Interface between Gold and Water: The Effect of Miniaturization and Roughness

Another critical factor is thermal coupling to the environment. We have already mentioned that thermal coupling between gold nanorods and their environment is a critical step in the photoacoustic process, because the photothermal conversion arises within the particles, but the thermoelastic expansion occurs in their dispersion medium. In practice this factor may be optimized either by lowering the thermal resistance at the interface between the particles and their dispersion medium or by increasing the specific surface area of the particulate.

The notion to purposely minimize the interfacial thermal resistance is a difficult one, because a rational engineering of phonon-phonon coupling at a heterogeneous boundary remains a tremendous challenge. The thermal resistance at the interface between gold particles and their aqueous environment was shown to vary between about 2 and 30 × 10${}^{-9}$ m${}^{2}$ K W${}^{-1}$, and to depend on a number of intrinsic parameters, such as wall temperature and heat flux [214], nanoscale pattern [215], radius of curvature [216] and wettability [217]. When extrinsic factors are incorporated in an effective definition of thermal resistance, such as underresolved features as local roughness or the presence of adlayers [218], such as surfactants or polymers used to stabilize a suspension of gold nanorods, but also silica itself [63,156,163,164], or the formation of vapor bubbles [219,220], its range of variability may span orders of magnitude. In particular, according to Chen et al [163], the effect of silica on the amplification of photoacoustic signals relates to an overall decrease of this parameter. Moon et al. [221] reported a similar effect in the case of gold nanorods coated with graphene oxide.

The idea to rationally maximize the specific surface area of the particulate is more feasible. One possibility may be to miniaturize the gold nanorods. To a first approximation, according to Gans theory [80], dividing one larger particle into N smaller particles with the same total volume and the same shape does not affect the optical absorbance and so the efficiency of photothermal conversion of the system. If those particles were isolated from their environment, the system made of N smaller individuals would undergo more deformation, because smaller gold nanorods are less stable and more prone to pre-melting, for the reasons outlined in Section 3.2. However, the thermal resistance at the interface between gold and aqueous is not infinite, and the system made of N smaller particles exhibits a specific surface area that is larger than that of their bigger peer by a factor $\sqrt[{3}]{N}$. It turns out that the advantage brought by a larger specific surface area typically more than compensates the loss of thermal stability in the miniaturized system, so that using smaller gold nanorods is an effective strategy to gain photostability.

Back in 2014, some of us demonstrated a striking gain in photostability upon miniaturization of gold nanorods [17]. A decrease in effective radius by a factor around 4 produced an increase in damage threshold exceeding a factor of 3, which is the outcome expected from an equivalent shell of silica as thick as around 30 nm [108]. By the help of FEM simulations, this effect was attributed to a major decrease of peak temperature reached within each particle, thanks to a better relaxation across a realistic model of thermally resistive boundary. Later authors have confirmed the same result [222] by the implementation of different protocols for particle miniaturization as well as different platforms for PAI, thus corroborating the robustness of this approach.

In a recent work, we have proposed an alternative concept to increase the interface contact area between the gold nanorods and their aqueous environment by the deposition and galvanic etching of a silver shell conveying a rough or porous gold overcoating [15]. We found that the threshold fluence for the onset of photoinstability almost scales with roughness, in spite of the fragility brought by the porous structure.

#### 4.2.1. Miniaturization of Gold Nanorods: Useful Protocols

**Figure 8:**
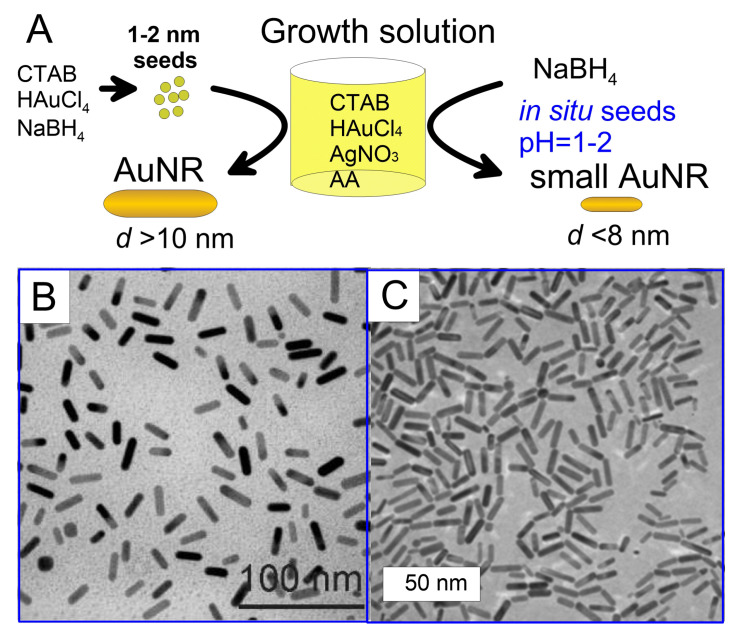
(**A**) General schemes of seed-mediated and seedless synthesis of small gold nanorods. (**B**) TEM image of small nanorods obtained by using cetyltripropylammonium bromide as stabilizing surfactant. Adapted with permission from Jia et al. [226], Copyright (2015) American Chemical Society. (**C**) TEM image of small gold nanorods obtained by using the so-called seedless approach. Adapted with permission from Ali et al. [227], Copyright (2012) American Chemical Society.

In the beginning, miniaturization has been pursued as a precondition to maximize the optical absorbance of gold nanorods per unit volume, and so their efficiency of photothermal conversion. The common seed-mediated synthesis gives particles with diameter larger than about 10 nm, and their cross sections for optical scattering are a minority but non-negligible fraction of those of optical extinction [77]. The most obvious recipe to reduce the size of gold nanorods is to increase the density of seeds added to the reaction mixture (Figure 8A) [17,223]. The rationale is that, if the addition of one seed leads to the growth of one particle, then an increase in the number of seeds, say, by a factor of 8 at same amount of Au(III) precursor should lead to an 8-fold decrease in particle volume, or two folds in diameter [17]. However, this approach does not only tend to produce a consistent decrease in particle size, but also modifies the overall growth kinetics and turns out to convey a larger amount of byproducts of undesirable shape [224]. In order to overcome this difficulty, Murphy and coworkers [224,225] suggested to achieve more control of all process parameters by using a millifluidic reactor. In some conditions, these authors reported rather thin gold nanorods with diameters of 6.6 nm at the gram-scale, but the possibility to combine yield and size control was still quite limited. Wang and coworkers obtained high quality small gold nanorods by combining a change in the molar ratio between seeds and Au(III) precursor with the use of cetyltripropylammonium bromide as a replacement to cetrimonium bromide as stabilizing surfactant [226]. When the seed density increased in a given growth solution, the size of the final gold nanorods decreased, and the minimum reported thickness was around 6 nm (Figure 8B).

El-Sayed and coworkers [227] developed a novel so-called seedless method for the synthesis of small gold nanorods (Figure 8A), which consists in a direct injection of NaBH${}_{4}$ into the reaction mixture for rapid initial nucleation. Strictly speaking, the word seedless is evocative but actually misleading, because it is not that seeding is missing, but it simply occurs in situ, rather than in a separate vial prepared in a preliminary step, as in their original protocol [84]. These authors found that pH plays a crucial role in the monodispersity of the particles. At very acidic conditions, i.e., pH around 1 to 2, the reducing power of ascorbic acid and NaBH${}_{4}$ decreases and the homogeneity of small gold nanorods happens to improve. As a result, high-quality gold nanorods were obtained with diameter as small as 5 nm (Figure 8C). In line with the most traditional protocols, the amount of added AgNO${}_{3}$ and NaBH${}_{4}$ controls the aspect ratio of the resulting particles. This seedless method has been further modified by the use of aromatic compounds [228] and by adjusting the reaction temperature until 97 ${}^{\circ }$C [229] to improve the possibility to tune the size of smaller rods.

Overall, both strategies based on increasing the dose of seeds or directly injecting NaBH${}_{4}$ into the reaction vial intend to modulate the competition between nucleation and growth in the synthesis of gold nanorods. However, this undertaking is intrinsically challenging, because the quality of the final sample depends on a subtle balance of kinetic conditions. Increasing the dose of seeds probably tends to provide more control and reproducibility [230]. But the seedless approach represents an attractive alternative in the overall picture, because the synthesis of seeds in the classical two-step protocol is a substantial and time-consuming complication that it would be convenient to remove anyway.

#### 4.2.2. Miniaturization of Gold Nanorods: Pros and Cons

As we have already mentioned in Section 3.2, the use of smaller particles, as well as any other solution to increase the specific surface area of the system thus departing from its Wulff construction, entails a lower thermal stability. Therefore, the regime of relevance of this strategy is subtle and presumes a strong competition between excitation and relaxation: in the case of too short or too long the optical pulses, the loss of thermal stability may prevail over the gain in heat transfer to the environment, and particle miniaturization may become counterproductive. The ns regime of most typical interest in PAI seems to be ideal. In addition, other considerations require a word of caution. A very practical downside of particle miniaturization is much more difficulty to implement the standard protocols for purification, modification etc. by the use of cycles of centrifugation and decantation, because pelletting smaller particles may amount to a time-consuming challenge with devices operating below 50,000 rcf. Moreover, in general, smaller particles tend to display broader plasmonic bands [73,231] for a mixture of intrinsic factors and problems related to the heterogeneity of the ensemble. The increase of specific surface area tends to favor the retention of cytotoxic impurities used in the synthesis, and so to elicit more cytotoxicity [230]. At a certain point, it is well known that gold clusters smaller than about 2 nm loose inertness and start to display catalytic behavior [232,233] that may impart various mechanisms of biological damage. Another item to keep in mind is that lower peak temperatures around individual particles may complicate certain niche applications, such as those based on the ignition of photocavitation [234].

However, this said, there also exist very cogent arguments in favor of particle miniaturization. In principle, accelerating the thermal coupling between gold nanorods and their environment does not only affect their photostability, but may also enhance the efficiency of photoacoustic conversion, because the source of pressure associated to the thermoelastic conversion in the suspension medium scales as the square of the time derivative of temperature. This effect was invoked in the case of silanized [163] as well as roughened [15] particles. Instead, the case of particle miniaturization may be more subtle, because a faster transient may compete with a lower peak temperature in their environment [15]. A decisive advantage of particle miniaturization is that this direction may help to conform to ADMET criteria and regulatory constraints. While we do not want to delve into the subtleties of this issue, it is important to mention that the pursuit of noble-metal particles with at least one cross section smaller than about 5 nm represents a hopeful target to gain compatibility with renal clearance [235,236,237], and so to temper their long-term accumulation. The biocompatibility of non-biodegradable nanoparticles smaller than about 5 nm may be a watershed discovery for the clinical use of gold nanorods. For the considerations made on the issue of cytotoxicity, the ideal window may be a narrow gate between about 2 to 5 nm.

Overall, particle miniaturization or any other way to enhance their rate of dissipative cooling is an effective tool to gain photostability for PAI. However, this solution is less universal than the concept to improve their thermal stability, and probably requires careful verification from case to case. In any way, we believe that particle miniaturization may be the lifeline for a clinical exploitation of gold nanorods, should this roadmap really prove to convey biocompatibility. Therefore, photostability may become an important side benefit for the translational development of this material.

Before we conclude this paragraph, we also point out that the concept of thermal coupling to the environment and its competition with a loss of thermal stability may arise also in contexts where an increase in specific surface area is not purposely targeted towards a gain in photostability. For instance, in a recent contribution [238], some of us found that longer-aspect ratio gold nanorods designed to resonate at lower frequencies enjoy no loss of photostability under a photoacoustic microscope, in spite of their further departure from Wulff construction, thanks to a faster relaxation.

### 4.3. Alternative Strategies

In previous sections, we believe that we have summarized the two most established highways to enhance the photostability of gold nanorods for PAI. Of course, there may also exist alternative or synergistic solutions. For instance, we suggest that another potential strategy may arise from the general notion to share the same extent of optical absorbance among more particles in the ensemble. The fact that the shape of gold nanorods is intrinsically anisotropic and typically inhomogeneous from particle to particle translates into a broad heterogeneity of photophysical behaviors. In practice, when monochromatic and linearly polarized light is used as optical trigger, energy is absorbed only by a small fraction of gold nanorods that enjoy near-resonant aspect ratio and proper alignment between their longitudinal axis and the electric field. In a recent publication [135], some of us estimated how close the resonant condition should be, and how small the subpopulation involved in the photoacoustic conversion ends up being, under typical conditions. The result was about 1/30. In particular, a factor 1/3 originates from the effect of anisotropy, and another factor around 1/10 from the inhomogeneity seen in a typical ensemble. These figures suggest two possible expedients to delay the onset of photodamage, i.e., the use of non-polarized or circularly polarized light, and non-monochromatic pulses with a bandwidth overlapping the plasmonic band of the ensemble. In particular, the use of non-polarized or circularly polarized light would redouble the fraction of resonant particles, and ideally leave unused only those aligned with the optical axis. Instead, the use of non-monochromatic light would allow recovering another factor as large as around 10. The combination of both solutions may improve the photostability by as much as about 20 folds, thus potentially representing the most impactful strategy among those outlined in this mini review. However, to the best of our knowledge, this strategy has never been explicitly proven, thus far. Instead, the effect of selective reshaping with monochromaticity and polarization has been reported in different contexts. For instance, a so-called phenomenon of hole burning [143,239] is often observed in the optical spectra of gold nanorods exposed to trains of short pulses, where the contribution from the resonant particles tends to disappear over time. In an interesting publication dating back to 2009, Zijlstra et al. [136] exploited the effects of polarization and monochromaticity, in combination with high spatial control, to selectively photodamage gold nanorods embedded in PVA films and demonstrate a simple device for optical data storage and readout over five dimensions.

An equivalent variant to this idea to consider the effect of inhomogeneous broadening in the particle ensemble, may be to enforce a concept of homogeneous broadening, where a smoother plasmonic band in each particle would result into more particles resonating with less intensity over the same monochromatic excitation. This effect may for instance concur in the case of particle miniaturization, where smaller particles tend to exhibit broader peaks of optical absorbance [73,231], and was explicitly invoked in the case of other models of plasmonic particles, such as gold nanoshells [60]. We expect that these possibilities will receive more attention in the near future.

## 5. Conclusions and Perspective

In this article, we have highlighted different approaches to enhance the photostability of gold nanorods for applications in photoacoustic imaging. Despite the numerous advantages of gold nanorods associated with their tunable plasmonic resonances and extensive possibility of bioconjugation, their practical use as contrast agents in photoacoustics is limited by their ease to undergo photodamage. The photoinstability of gold nanorods is not only a phenomenon of academic interest, but also a concern of very practical relevance. Gold nanorods tend to lose efficiency of photoacoustic conversion upon exposure to ns-long pulses well before 10 mJ/cm${}^{2}$, which is below relevant permissible exposure limits for most cases of interest. As a quantitative measure of photoinstability, the drop of optical absorbance at the plasmon resonance wavelength is usually used for its high sensitivity to the structural processes of spheroidization or destruction. However, the most convenient approach is based on a direct assessment of the drop of photoacoustic signal, since it combines both diagnostic value and practical significance.

We have tried to classify the strategies used to increase the photostability of gold nanorods according to few basic principles. First, the use of rigid or soft shells is arguably the most popular solution implemented to constrain the initial shape and inhibit the progress of premetling. The use of silica or polymer coatings of a thickness in the order of several tens of nanometers increases the photodamage thresholds of gold nanorods by several folds. Another useful approach relates to lowering the thermal resistance at the interface between gold and water, which may be achieved by a decrease of average size or, for instance, an increase of roughness. In particular, particle miniaturization easily conveys an effect of an extent similar to that of silica coatings. Finally, we suggest that biggest advantages may be achieved by shifting the focus to the optical source, in order to redistribute the input energy among more particles by delivering non-polarized and non-monochromatic light.

While more ideas are expected to unfold in the years to come, the combination of these solutions is on track to convey the due compliance with the conditions in use in preclinical and clinical systems for photoacoustic imaging. We are confident that our work will inspire new research towards a broader use of gold nanorods for applications in photoacoustics.

## Figures and Tables

**Table 1:** Commercial photoacoustic platforms and their relevant features. An (*) highlights the optical specifications of more direct concern to the photostability of the contrast agent.

Device	MSOT Acuity [19]	Vevo LAZR-X [20]	LOIS3D [21]	TriTom [22]	Hadatomo Z [23]
Company	iThera Medical	FUJIFILM	TomoWave	PST	Advantest Corp.
Pulse duration *	<10 ns	4–6 ns	3–5 ns	5 ns	10 ns
Rep rate *	25 Hz	20 Hz	10 Hz	10 Hz	1000 Hz
Wavelength *	660–1300 nm	680–970; 1200–2000 nm	680–1064 nm	670–2600 nm	532; 556 nm
Peak energy *	30 mJ	45 mJ	up to 200 mJ	150 mJ	16 μJ
Imaging depth	up to 4 cm	1 cm	>3 cm	n.a.	3 mm
Lateral resolution	200 μm	45 μm	250 μm	150 μm	15 μm
Applications	clinical	pre-clinical	pre-clinical	pre-clinical	clinical

**Table 2:** Endogenous contrast agents for PAI (NIR-I = 650–1000 nm; NIR-II = 1000–1700 nm [32]).

Chromophore	Wavelenght Window	Clinical Interest
Melanin	NIR-I and NIR-II	Skin cancer
Hemoglobin (Oxy-Deoxy)	NIR-I	Ischemia, hypoxia or hypoxemia, tumor angiogenesis
Lipids [33]	NIR-II	Arterial plaques monitoring, diabetes, obesity, fatty liver disease
Collagen [34]	NIR-II	Orthopedics, dermatology, and cardiology

## Data Availability

The data are available on request from the corresponding author.
